# Rehabilitation of extremely atrophic edentulous mandible in elderly patients with associated comorbidities: a case report and proof of concept

**DOI:** 10.1186/s13005-021-00274-2

**Published:** 2021-06-29

**Authors:** Iulian Filipov, Lucian Chirila, Corina Marilena Cristache

**Affiliations:** 1 “Queen Maria” Military Emergency Hospital, 9 Pietii Str, 500007 Brasov, Romania; 2 Department of Oral and Maxillofacial Surgery, Faculty of Dental Medicine, “Carol Davila” University of Medicine and Pharmacy, 19 Plevnei Ave, 010221 Bucharest, Romania; 3 Department of Dental Techniques, Faculty of Midwifery and Medical Assisting (FMAM), “Carol Davila” University of Medicine and Pharmacy, 8, Eroilor Sanitari Blvd, 050474 Bucharest, Romania

**Keywords:** Atrophic mandible, Bar overdenture, Dental implants, Bypassing alveolar nerve

## Abstract

**Background:**

Oral rehabilitation of the atrophic mandible is, most of the time, a challenging procedure, especially in elderly patients with associated comorbidities.

**Case presentation:**

This clinical report describes the rehabilitation of an extremely atrophic mandible using an overdenture supported by four splinted implants, two of which are placed in the interforaminal region and the other two bypassing the inferior alveolar nerve at the level of the antegonial notch. A passive-fit bar structure splinting the four inserted implants was designed to compensate for mandibular flexure, to reduce the amount of strain on the implants, and avoid bone resorption and prosthetic failure. The 14-month postoperative cone-beam computed tomography (CBCT) and the clinical follow-up showed the bilateral integrity of the inferior alveolar nerve and the successful restoration of the atrophic edentulous mandible with a significant improvement in the patient’s quality of life.

**Conclusions:**

The applied technique depicts several benefits such as a minimally invasive approach, reduced number of surgical interventions, reduced total treatment time, reduced treatment costs, and higher psychological acceptability.

## Background

As the absence of teeth is frequent in elderly people and the volume of alveolar bone is dictated by the presence of teeth, many of these patients exhibit significant bone loss that might lead to functional limitations, aesthetic worsening, psychological impairment, and social limitations. In many cases, elderly people wearing complete dentures have to deal with pronunciation and mastication difficulties especially due to lack of denture retention. Moreover, improper nutrition may significantly affect their general health.

The objectives of dental health rehabilitation for elderly patients are to ensure a good dental quality of life, maintain self-esteem, and facilitate proper nutrition without adding problems that could adversely affect their daily lives.

The presence of chronic diseases, polypharmacy, age-associated changes in oral tissues, as well as the socioeconomic characteristics of the aging population are important challenges in treatment planning [1].

Moreover, prosthetic restoration of the severely resorbed edentulous mandible is challenging due to the pattern of bone resorption and the presence of the alveolar nerve [2]. Compared to a maxillary denture, a mandibular denture has a base with a longer periphery and smaller contact area between the alveolar ridge and the denture base; fixed mucosa are also frequently lacking. These anatomical features may have a negative influence on the successful use of a mandibular denture, causing instability due to insufficient retention, acute pain by overloading the mucosa, or even worse, by compressing the mental nerve, impaired masticatory function, speech difficulties, loss of soft tissue support, altered facial appearance, psychological impairment, and social limitations [3, 4].

To improve retention and stability, the two-implant overdenture has become the first choice of treatment for the edentulous mandible [5–7]. However, with the insertion of two interforaminal implants, mandibles with a height of less than 10 mm, as measured at the symphysis, are at risk of fractures and associated complications [8, 9].

Several surgical options are also envisaged: Augmentation procedures could be an option for restoring the alveolar bone volume but major bone grafting techniques of extremely resorbed mandibles may not be justified in cases where shorter implants could be placed [10]. Moreover, medically compromised patients would not be appropriate candidates for major augmentation procedures due to the high risk of complications. Transposition of the mandibular nerve is a possibility in cases with sufficient bone both below and above the mandibular canal. However, permanent paraesthesia of the inferior alveolar nerve is a frequently reported complication [11].

In certain cases, short implants [12] or tilted implants with a bicortical anchorage placed in the premolar and molar areas, bypassing the inferior alveolar nerve, could be a treatment option [13].

Titanium subperiosteal implants with simultaneous bone morphogenetic proteins (BMPs) graft have been proposed for atrophic mandibular bone preservation and overdenture retention [14]. However, this surgical procedure is invasive and subperiosteal implants are prone to long-term complications [15, 16].

A successful mandibular rehabilitation with overdentures supported by two splinted or unsplinted interforaminal endosteal implants, in cases of extreme alveolar bone resorption is limited by the risk of mandible fracture at the time of implant placement or after occlusal loading [17].

For patients with atrophic mandible and associated comorbidities such as diabetes mellitus and ischemic heart disease, accomplishing rehabilitation of the full mandibular arch requires a minimally invasive approach and can be a challenging goal.

The aim of this paper is to present a minimally invasive treatment option for the rehabilitation of extremely atrophic mandible using a bar-retained overdenture supported by four splinted implants, two placed in the interforaminal region and the other two bypassing the inferior alveolar nerve at the antegonial notch level.

## Case presentation

**Fig. 1:**
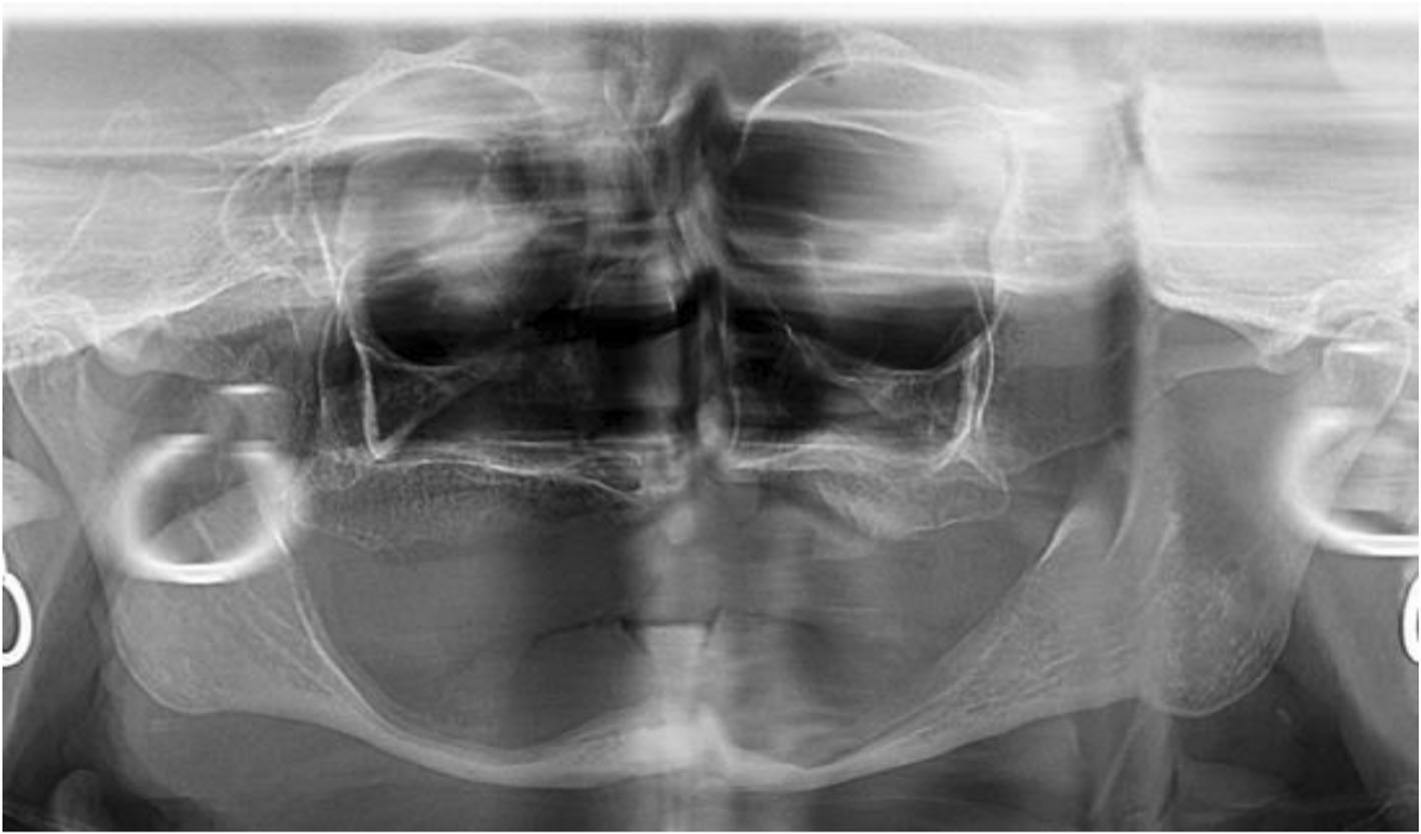
Preoperative orthopantomography

A 72-year-old female, fully edentulous, wearing a removable prosthesis, was referred to our clinic after three unsuccessful attempts at rehabilitation of the edentulous mandible, with complete dentures. The patient had a 16-month history of swallowing problems, a painful erosion lesion, burning sensations on the left floor of the mouth, and recurrent numbness in the lower-right inferior lip. The X-ray evaluation showed extreme resorption of the mandible, as seen in Fig. 1.

The patient was content with her maxillary denture, which was satisfactory from a technical point of view.

Her medical history revealed type 2 diabetes mellitus managed with medication, psychological depression, class II obesity, obstructive sleep apnea, class III (moderate) to IV (severe) heart failure according to NYHA (New York Heart Association) classification, controlled essential hypertension, and ischemic heart disease.

The preoperative CBCT confirmed resorption of the alveolar bone and partial resorption of the basal bone, a residual bone height between 5 and 8 mm corresponding to the interforaminal region, and the exposure of the inferior alveolar nerve (Class V-VI according to Cawood and Howell classification [18]), with no residual bone above the nerve in the lateral region (between the mental foramen and the second molar).

After presenting all the available options, including mental nerve transposition and extensive bone grafting, the accepted and less-invasive treatment was an overdenture supported by four splinted dental implants, without any bone grafting. The insertion of only two interforaminal implants was excluded due to the high risk of mandibular fracture. Therefore, in the third molar region, the inferior alveolar nerve by-pass technique was considered to be appropriate in this case. Prophylactic antibiotic therapy was initiated within one hour before surgery; the patient was administered with 2 g of a combination of amoxicillin and potassium clavulanate.

Mepivacaine HCl 3 % without vasoconstrictor (Scandonest 3 %, Septodont, Saint-Maur-des-Fossés, France) was locally infiltrated into the lingual and the labial aspect for each implant site. A full muco-periosteal flap was elevated to obtain direct visual access to the residual bone.

**Fig. 2:**
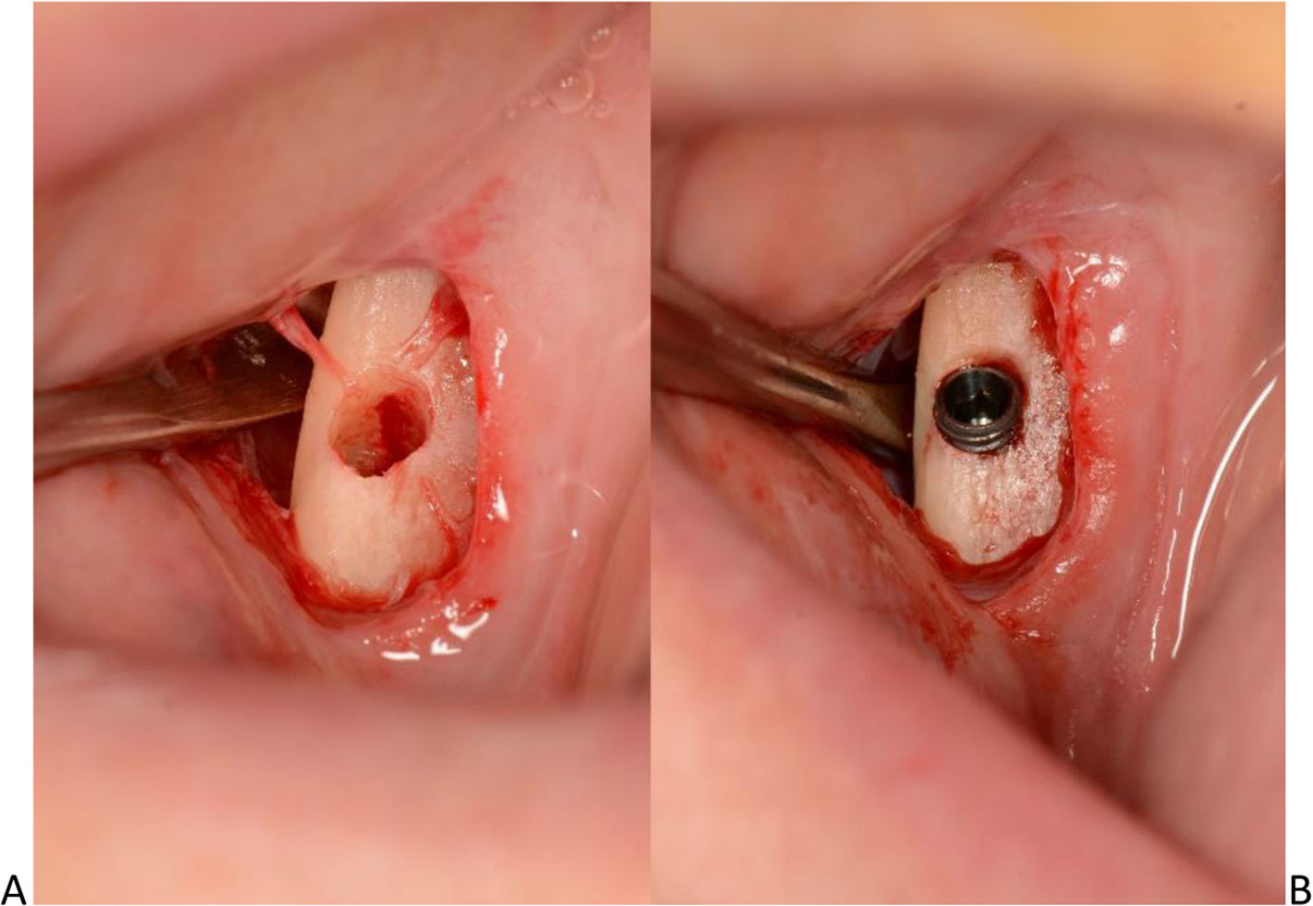
**A**. Osteotomy for dental implant insertion at the retromolar area. The alveolar nerve was bypassed at the buccal side. **B**. The dental implant was inserted

The osteotomies were performed at 300 rpm and 50Ncm under cold saline irrigation using a freehand technique. In the interforaminal region, two 3.5 mm x 7mm implants (AnyRidge®, MegaGen, Daegu, Korea) were placed with an insertion torque of 40Ncm (right) and 45 Ncm (left), respectively. In the third molar area, two 3.2 mm x 10 mm implants (Mini®, MegaGen, Daegu, Korea) were placed with an insertion torque of 35 Ncm. The implants were inserted in the residual buccal bone bypassing the inferior alveolar nerve, as shown in Fig. 2A and B.

The healing abutments were installed immediately: two conventional in the anterior region and two custom made; 12 mm in length healing in the posterior mandible. Simple interrupted sutures were used to close the incisions. 2 g of amoxicillin and clavulanate potassium were administered for the following 4 days. Sensory condition in the lower lip and chin was evaluated 24 h after the surgery, and no neurosensory changes were present. Healing was uneventful and the sutures were removed 14 days after surgery. Restorative treatment was initiated at 22 weeks post-implant insertion and the patient received the final overdenture 22 days after the preliminary impression.

**Fig. 3:**
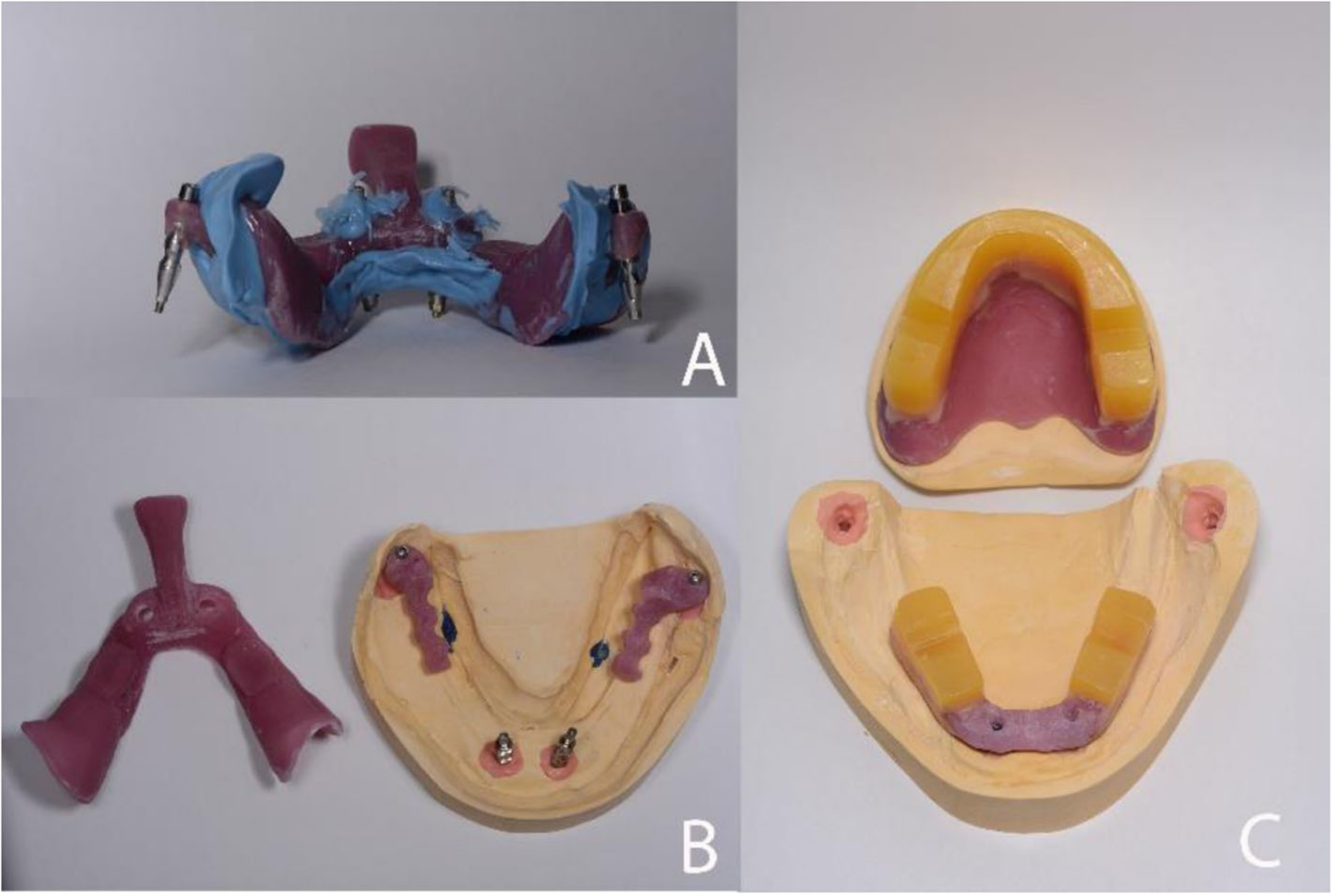
**A**. Functional impression. **B**. The three-part custom tray was used, for an accurate functional impression, due to the posterior implant positioning and limited mouth opening. **C**. Mandibular and maxillary record basis and occlusal rims

**Fig. 4:**
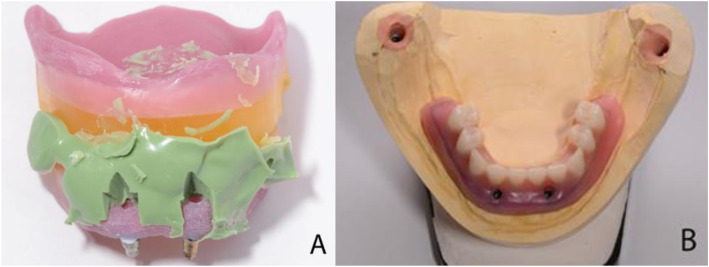
**A**. Centric relation (CR) registration. **B**. Try-in mandibular denture. The mandibular denture was limited to the two premolars due to an inadequate intermaxillary hight

Due to the high level of mandibular atrophy, a special three-part customized impression tray was made as shown in Fig. 3A and B. The corresponding transfer abutments, AnyRidge®, for the two interforaminal implants, and Mini®, for the posterior implants, were inserted and functional impressions were taken with a polyether material (Impregum; 3 M ESPE, St. Paul, MN, USA), as shown in Fig. 3A. The three-part customised tray was made on a preliminary cast with implant analogues poured from a preliminary impression with the corresponding transfer abutments. Jaw relations were recorded with record bases and occlusal rims as shown in Figs. 3 and 4A and C.

An Artex face bow (Amann Girrbach, Koblach, Austria) was used to transfer the horizontal relationship of the maxillary arch to the cranial base and data were employed to mount maxillary and mandibular casts in an Artex®CR (Amann Girrbach AG, Koblach, Austria) -Arcon articulator, following the manufacturer’s instructions. A try-in mandibular denture was manufactured and the required functional adjustments were performed, as shown in Fig. 4B. A removable bar-retained overdenture was planned. Four OT Equator® abutments (Rhein83, Bologna, Italy) were screwed onto the implants, two custom-made 11 mm abutments on the posterior implants and two 4 mm abutments in the interforaminal region.

**Fig. 5:**
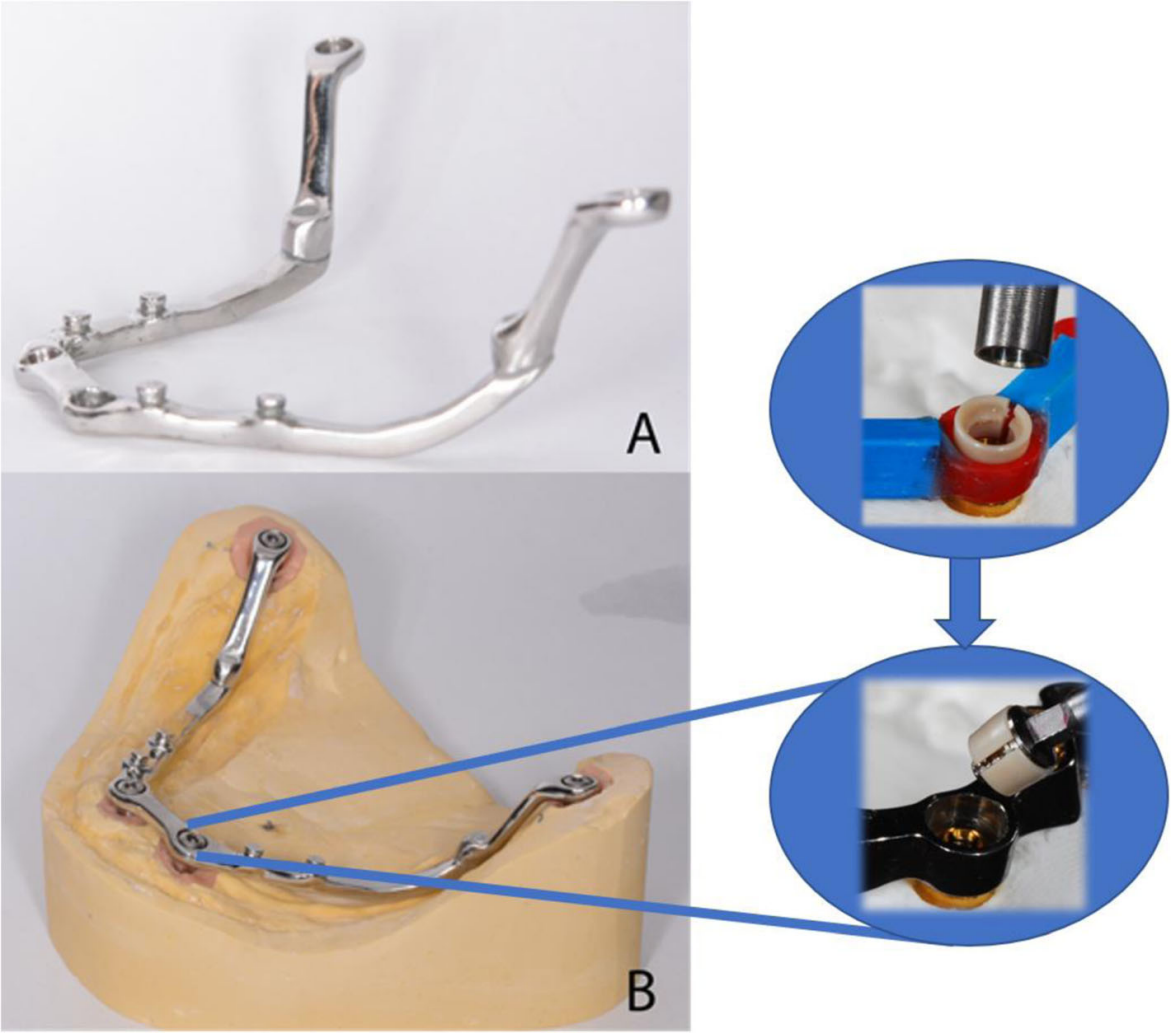
**A**. The custom-made titanium Seeger bar. **B**. The titaium bar screwed on the OT Equator® analogues on the functional cast. The Seeger bar was obtained from castable components: container for cilinder Seeger ring (red color) and castable conector (blue). The white self-extracting Seeger ring is inserted on the Equator abutment before applying the titanium locking screw

A custom-designed bar secured with four castable Seeger Bar containers (Rhein83, Bologna, Italy), as shown in Fig. 5A and B was screwed over the OT Equator® abutments, with titanium locking screws and self-extracting Seeger rings (Rhein83, Bologna, Italy). The bar’s link framework was made with castable components: OT Bar gingival connectors. To anchor the over-structure, four single OT Equator® castable retentions (Rhein83, Bologna, Italy) were placed balanced distributed on the canine-premolar regions of the bar to ensure polygonal support for the denture. Both the bar and link frameworks were cast from Cr-Co alloy, together in the same duplicating mould to equalise the volumetric changes for both structures. After casting and postprocessing, the link framework was bonded to the final denture using a light-curing resin.

**Fig. 6:**
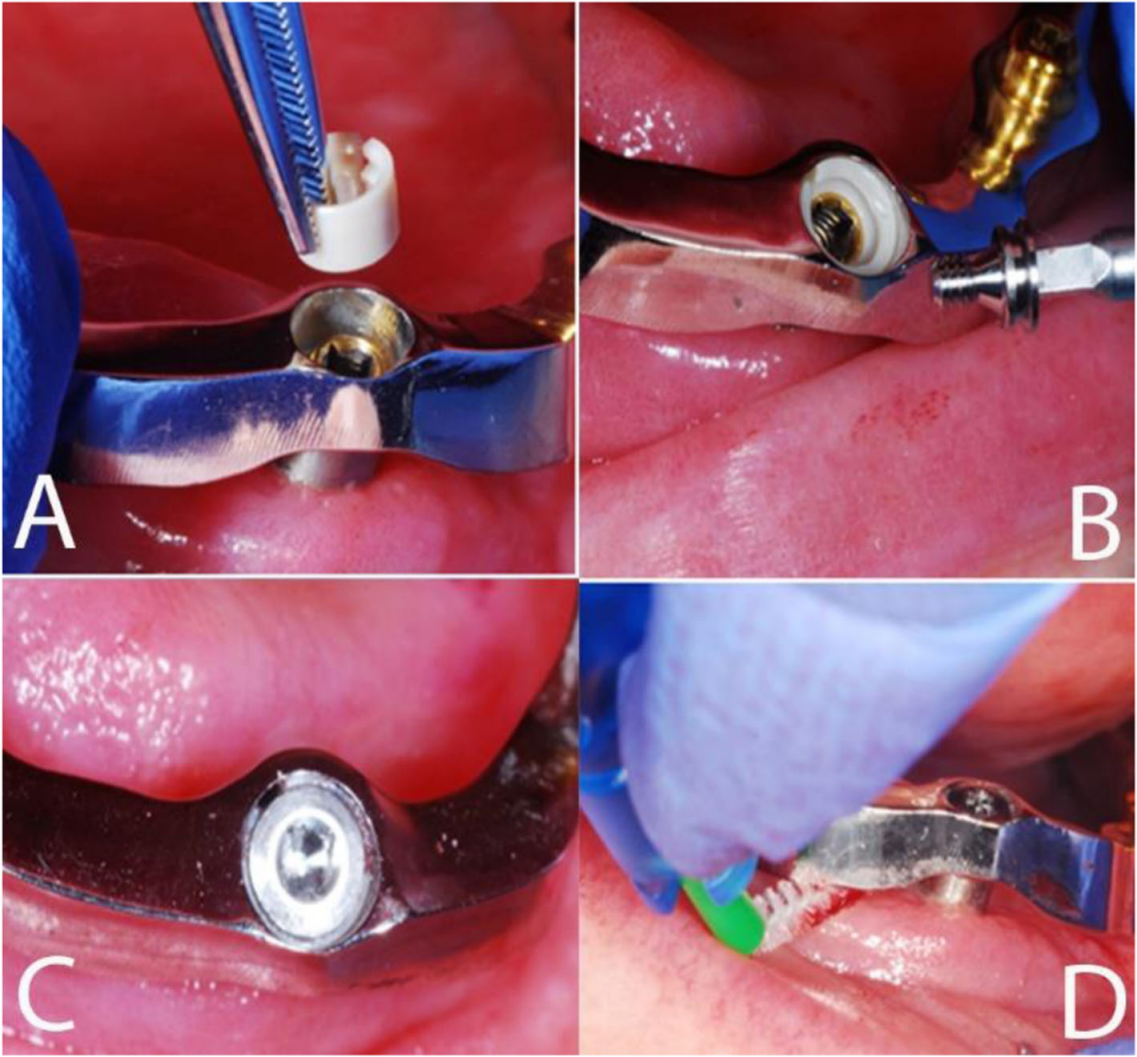
Insertion of the bar over OT Equator® abutments. **A** Seeger ring is applied over the abutment upon bar insertion; **B** The titanium screw is screwed on the abutment to retain the bar; **C** Occlusal view of the screwed bar; **D** Adequate space was designed between the bar and the underlying mucosa for a good oral hygiene

The bar was mounted on the four OT Equator® abutments by using the self-extracting elastic Seeger rings over the abutments, with the aim to obtain a passive structure and avoid stress distribution to the dental implants (Fig. 6A-C). Also, the space between the bar and the underlying mucosa was designed adequately for oral hygiene maintenance (Fig. 6D).

**Fig. 7:**
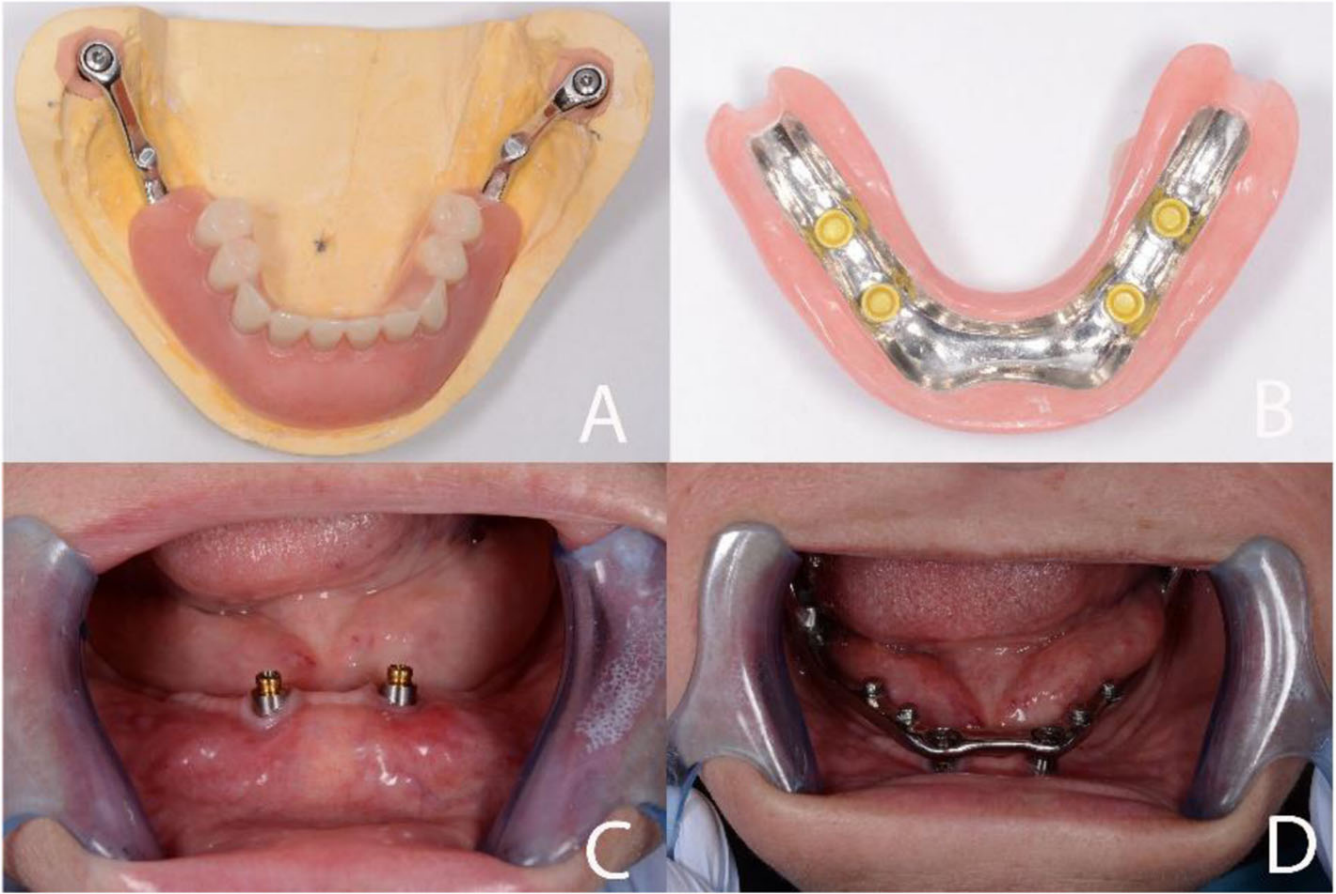
**A**. The final overdenture applied to the titanium bar. **B**. The intaglio surface of the overdenture with yellow extra-soft retentive caps applied. **C**. OT-Equator abutments screwed onto the implants. **D**. Seeger titanium bar inserted

Due to the pattern of atrophy, the distal parts of the bar were not covered by the mandibular overdenture, as shown in Fig. 7A-D.

**Fig. 8:**
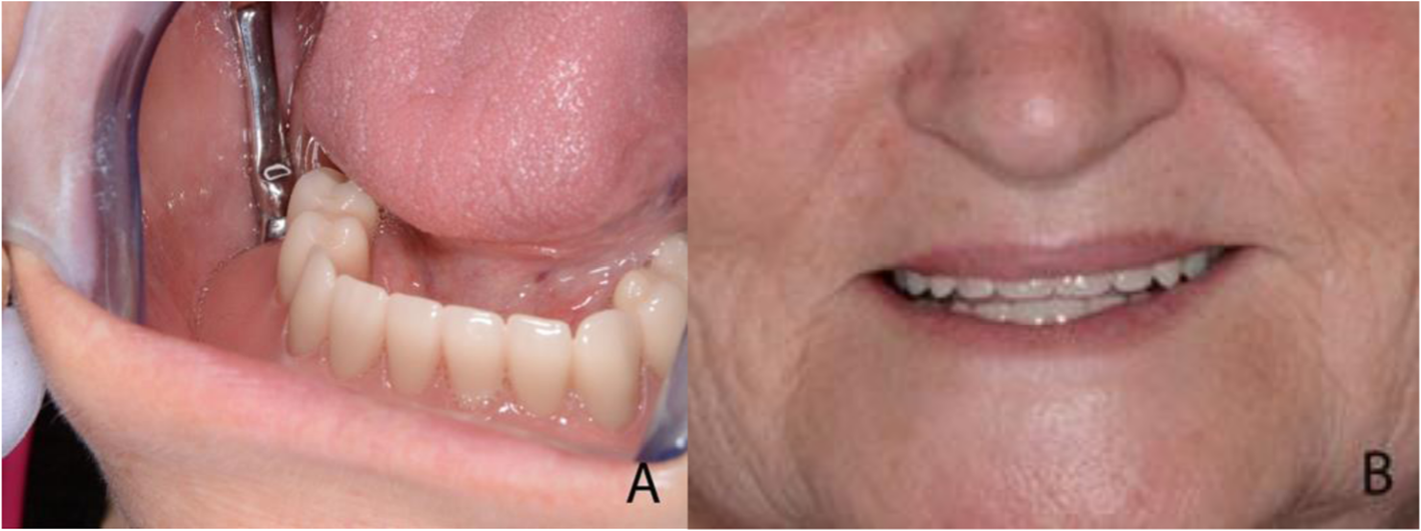
**A** Right-side intraoral view with the bar overdenture in place. **B**. The patient with the mandibular overdenture after functional adjustments

**Fig. 9:**
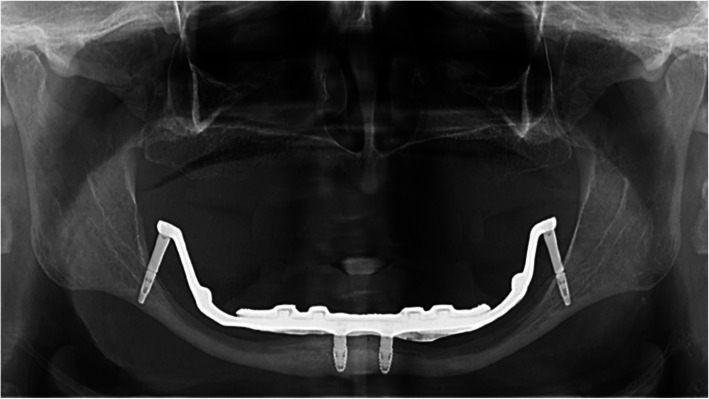
Post denture insertion orthopantomograpy

At the delivery of the denture, functional adjustments were performed, and soft retention Nylon inserts were used. Oral hygiene instructions, including the use of interproximal brushes and oral irrigators, were provided to the patient. The patient was extremely happy with the functional and aesthetic outcomes, as shown in Fig. 8A and B. The correct positioning of the bar was assessed with the aid of an orthopantomography, as shown in Fig. 9.

At one week post denture insertion follow-up, the patient had normal lower-lip sensitivity, improved masticatory ability, and normal deglutition.

The patient’s self-perception in relation to the impacts of oral conditions on physical, psychological, and social wellbeing was evaluated before treatment and one week post mandibular overdenture insertion using the Oral Health Impact Profile for Edentulous Patients (OHIP‐EDENT) questionnaire, validated for the Romanian language (ClinicalTrials.gov Identifier: NCT01392456). Each of the 19 items were assessed on a Likert scale (4 = always, 3 = frequently, 2 = sometimes, 1 = seldom, and 0 = never) with a total range of 0–76, a higher score meaning poorer quality of life [19]. Despite the good fit of the maxillary denture, the registered OHIP-EDENT total score was 69 prior to the treatment; however, it decreased to 19 with the final mandibular overdenture, showing a significant improvement in quality of life.

**Fig. 10:**
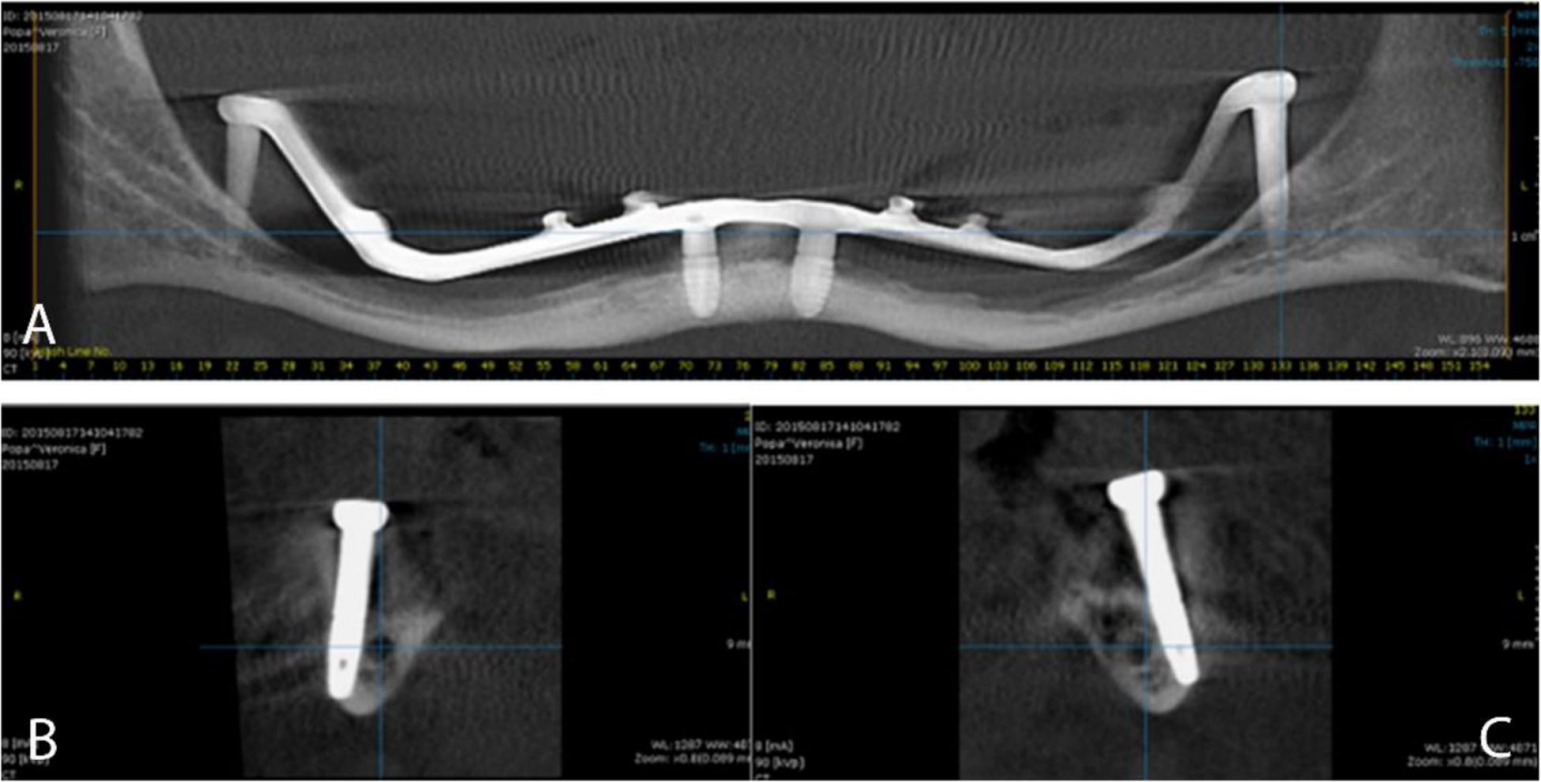
CBCT at 14 months follow-up. **A** Coronal view of the mandible with implants inserted and attached bar structure; **B** Sagital right and **C** Sagital left sections. The residual bone integrity and the absence of resorbtion could be observed in all sections

At the 14-month follow-up, the overdenture was evaluated and a CBCT taken, as shown in Fig. 10. The inferior alveolar nerve integrity and the stability of the bone tissue surrounding the dental implants were also assessed.

## Discussion and conclusions

The literature is scarce on previous reported cases of extreme atrophic edentulous mandible treated with bar overdentures on dental implants bypassing the inferior alveolar nerve.

This clinical case describes a successful technique of oral rehabilitation of an edentulous lower jaw with minimum residual bone in a patient with multiple comorbidities by means of an overdenture supported by four splinted implants, two of which bypass the inferior alveolar nerve at the level of the antegonial notch.

The number of implants and prosthesis design were intended to restore the resistance of the mandible to the occlusal forces and bending moments occurring during function. The designed bar showed an improved fitting over the inserted implants due to the circular container sitting over the OT Equator® abutment (Fig. 5B). The elastic, conical-shaped Seeger rings with vertical cut were placed at the bar-abutment junction, before screwing the bar to compensate for minor imprecisions during bar manufacturing [20].

The choice of the present treatment procedure was based on the necessity to reduce patient discomfort, especially the recurrent numbness in the lower-right inferior lip associated with swallowing problems, using a minimally invasive treatment path with a high level of predictability.

Dental implant rehabilitation of the edentulous mandible with the use of a bar structure and a removable prosthesis is considered a predictable, safe, and accessible technique for improving patients’ aesthetics and function [21]. The optimal number of implants for preventing complications has been discussed in numerous studies [22]. Cicciù et al., in a finite element (FEM) study on stress distribution over mandibular bone due to a four and six-implant-supported bar overdenture found that the relative reduction of stress on bone structures does not justify the use of a six implants [23]; at least two of the implants need to be installed in the posterior part of the dental arch. However, the anatomical condition of the jaws and the atrophic bone in the present case meant that a maximum of two implants could be inserted in the interforaminal region.

OT Equator® abutments were screwed onto the inserted implants to retain the bar structure. In a recent FEM study comparing three types of abutments (universal, Locator®, and Equator®) for overdentures, Cicciù et al. highlighted a better stress distribution over the underlying bone offered using the Locator® and Equator® systems, compared to a universal abutment [24]. Moreover, the Equator® system proved to involve less strain on the bone and peri-implant tissues; its shape, according to the results of the FEM study, appeared to collect the strength over the head of the retainer, favouring the higher stress on the retainer gum [24].

Due to the requirement of splinting the four inserted implants to avoid mandibular fracture, the passive-fit of the bar structure, obtained by using the elastic Seeger, was mandatory to compensate for mandibular flexure, which consequently reduces the amounts of strain on the implants and avoids bone resorption and prosthetic failure [25].

The elastic Seeger rings are inserted onto the OT Equator® of each abutment (Fig. 6A); according to Cicciù et al.’s FEM study [24], this will manage the higher stress, preventing the risk of mandible fracture during functional loading.

A fixed dental prosthesis on four implants could be a possible alternative to the mandibular bar-retained overdenture. However, a rigid structure is often associated with higher marginal bone loss, high frequency of complications, and poor plaque control, particularly in extremely atrophic bone [26]. A computer-aided design/computer-aided manufacturing (CAD/CAM) titanium bar [27, 28] was not considered an option in the present case, due to the unusual design required for the bar structure determined by the insertion of the posterior implants.

The applied technique depicts several benefits such as offering a minimally invasive approach, reduced number of surgical interventions, reduced total treatment time, reduced treatment costs, and higher psychological acceptability.

The presented protocol of atrophic mandibular rehabilitation with four implants inserted, two anterior and two in the retromolar area and the use of a passive bar without cantilever, could be a viable option for restoring functions and improving oral-health-related quality of life.

However, an experienced surgical and restorative team, a CBCT investigation and precise planning, the existing residual bone volume, the degree of mouth opening, the amount of mandibular atrophy and natural flexure, as well as the adjacent soft tissues, are important factors to be considered.

No complications occurred during the 14-month follow-up, demonstrating a successful surgical and restorative option.

## Data Availability

All data generated or analysed during this study are included in this published article.

## Abbreviations

BMPs: Bone morphogenetic proteins
CAD/CAM: Computer-aided design/computer-aided manufacturing
CBCT: Cone-beam computerized tomography
FEM: Finite element method
NYHA: New York Heart Association
OHIP-EDENT: Oral Health Impact Profile for Edentulous Patients

## References

[1] Dudley J (2015). Implants for the ageing population. Aust Dent J.

[2] Gonçalves F, Campestrini VLL, Rigo-Rodrigues MA, Zanardi PR (2020). Effect of the attachment system on the biomechanical and clinical performance of overdentures: A systematic review. J Prosthet Dent.

[3] Carlsson GE, Omar R (2010). The future of complete dentures in oral rehabilitation. A critical review. J Oral Rehabil.

[4] Limpuangthip N, Somkotra T, Arksornnukit M (2018). Modified retention and stability criteria for complete denture wearers: a risk assessment tool for impaired masticatory ability and oral health-related quality of life. J Prosthet Dent.

[5] Feine JS, Carlsson GE, Awad MA, Chehade A, Duncan WJ, Gizani S (2002). The McGill consensus statement on overdentures. Mandibular two-implant overdentures as first choice standard of care for edentulous patients.. Gerodontology.

[6] Cristache CM, Ionescu C, Burlibaşa M, Cristache G, Iliescu AA, Dumitriu HT (2009). Retentive anchors versus magnets as attachment systems for mandibular overdenture. A 5-year prospective randomised clinical study. Metal Int.

[7] Cristache CM, Ionescu C, Cristache G, Ionescu I, Iliescu AA, Burlibaşa M (2009). A 5-year prospective randomised clinical trial on the efficiency of two different attachment systems as retention for implant-supported mandibular overdenture. radiographic assessment, cost analysis and final evaluation of treatment’s success. Metal Int.

[8] Bender CA, Koudstaal MJ, Wolvius EB, Van der Kolk - (2018). Treatment of Severely Atrophic Edentulous Mandible Fractures: Load-Bearing or Load-Sharing?. Craniomaxillofac Traum Reconstr Open.

[9] Soehardi A, Meijer G, Stoelinga P (2017). An inventory of mandibular fractures associated in atrophic edentulous mandibles. Int J Oral Maxillofac Surg.

[10] Esposito M, Gabriella Grusovin M, Felice P, Karatzopoulos G, Worthington HV, Coulthard P (2009). The efficacy of horizontal and vertical bone augmentation procedures for dental implants— a Cochrane systematic review. Eur J Oral Implant.

[11] Vetromilla BM, Moura LB, Sonego CL, Torriani MA, Chagas OL (2014). Complications associated with inferior alveolar nerve repositioning for dental implant placement: A systematic review. Int J Oral Maxillofac Surg.

[12] Grant BTN, Pancko FX, Kraut RA (2009). Outcomes of Placing Short Dental Implants in the Posterior Mandible: A Retrospective Study of 124 Cases. J Oral Maxillofac Surg.

[13] Krekmanov L (2000). Placement of posterior mandibular and maxillary implants in patients with severe bone deficiency: a clinical report of procedure. Int J Oral Maxillofac Implants.

[14] Loperfido C, Mesquida J, Lozada JL (2014). Severe mandibular atrophy treated with a subperiosteal implant and simultaneous graft with rhbmp-2 and mineralized allograft: A case report. J Oral Implantol.

[15] Zwerger S, Abu-Id MH, Kreusch T (2007). Long-term results of fittig subperiosteal implants: report of twelve patient cases. Mund Kiefer Gesichtschirurgie.

[16] Schou S, Pallesen L, Hjørting-Hansen E, Pedersen CS, Fibæk B (2000). A 41-year history of a mandibular subperiosteal implant. Clin Oral Implants Res.

[17] Raghoebar GM, Stellingsma K, Batenburg RH, Vissink A (2000). Etiology and management of mandibular fractures associated with endosteal implants in the atrophic mandible. Oral Surg Oral Med Oral Pathol Oral Radiol Endod.

[18] Cawood JI, Howell RA (1988). A classification of the edentulous jaws. Int J Oral Maxillofac Surg..

[19] Cristache CM, Totu EE, Iorgulescu G, Pantazi A, Dorobantu D, Nechifor AC, et al. Eighteen Months Follow-Up with Patient-Centered Outcomes Assessment of Complete Dentures Manufactured Using a Hybrid Nanocomposite and Additive CAD/CAM Protocol. J Clin Med. 2020;9:324. Available from: https://www.mdpi.com/2077-0383/9/2/324. 10.3390/jcm9020324 PMC7073708 31979345

[20] Montanari M, Bonato G, Ortensi L (2016). Oral Rehabilitation with Implant-Supported Overdenture and a New Protocol for Bar Passivation. Glob J Oral Sci.

[21] Lauritano F, Runci M, Cervino G, Fiorillo L, Bramanti E, Cicciù M (2016). Three-dimensional evaluation of different prosthesis retention systems using finite element analysis and the Von Mises stress test. Minerva Stomatol.

[22] Roccuzzo M, Bonino F, Gaudioso L, Zwahlen M, Meijer HJA (2012). What is the optimal number of implants for removable reconstructions? A systematic review on implant-supported overdentures. Clin Oral Implants Res Suppl.

[23] Cicciù M, Cervino G, Milone D, Risitano G (2018). FEM investigation of the stress distribution over mandibular bone due to screwed overdenture positioned on dental implants. Materials (Basel).

[24] Cicciù M, Cervino G, Milone D, Risitano G (2019). FEM analysis of dental implant-abutment interface overdenture components and parametric evaluation of Equator® and Locator® prosthodontics attachments. Materials (Basel).

[25] Law C, Bennani V, Lyons K, Swain M (2012). Mandibular Flexure and Its Significance on Implant Fixed Prostheses: a Review. J Prosthodont.

[26] Tallarico M, Xhanari E, Kadiu B, Scrascia R (2017). Implant rehabilitation of extremely atrophic mandibles (Cawood and Howell Class VI) with a fixed-removable solution supported by four implants: One-year results from a preliminary prospective case series study. J Oral Sci Rehabilit.

[27] Pozzi A, Tallarico M, Moy PK (2016). Four-implant overdenture fully supported by a CAD-CAM titanium bar: A single-cohort prospective 1-year preliminary study. J Prosthet Dent.

[28] Tallarico M, Cervino G, Scrascia R, Uccioli U, Lumbau A, Meloni SM (2020). Minimally Invasive Treatment of Edentulous Maxillae with Overdenture Fully Supported by a Cad/Cam Titanium Bar with a Low-Profile Attachment Screwed on Four or Six Implants: a Case Series. Prosthesis.

